# The paired A–Not A design within signal detection theory: Description, differentiation, power analysis and application

**DOI:** 10.3758/s13428-021-01728-w

**Published:** 2022-02-07

**Authors:** Nina Düvel, Reinhard Kopiez

**Affiliations:** Hanover Music Lab, Hanover University of Music, Drama and Media, Neues Haus 1, 30175 Hanover, Germany

**Keywords:** Auditory perception, Signal detection theory, Statistical power, A-Not A design, Yes-no task, Music

## Abstract

Signal detection theory gives a framework for determining how well participants can discriminate between two types of stimuli. This article first examines similarities and differences of *forced-choice* and *A–Not A* designs (also known as the *yes-no* or *one-interval*). Then it focuses on the latter, in which participants have to classify stimuli, presented to them one at a time, as belonging to one of two possible response categories. The A–Not A task can be, on a first level, *replicated* or *non-replicated*, and the sub-design for each can be, on a second level, either a *monadic*, a *mixed*, or a *paired* design. These combinations are explained, and the present article then focuses on the both the non-replicated and replicated paired A–Not A task. Data structure, descriptive statistics, inference statistics, and effect sizes are explained in general and based on example data (Düvel et al., 2020). Documents for the data analysis are given in an extensive online supplement. Furthermore, the important question of statistical power and required sample size is addressed, and several means for the calculation are explained. The authors suggest a standardized procedure for planning, conducting, and evaluating a study employing an *A–Not A* design.

Many empirical studies in psychology and related disciplines try to determine how well participants can discriminate between two types of stimuli or how well they can allocate a stimulus to the correct stimulus class. Signal detection theory (SDT) was developed as an appropriate methodology to answer these questions based on empirical data. In the early 1950s, electrical engineers developed SDT (Swets, 1996, p. vii), and one of the first and central publications of the theory relating to the field of psychophysics was published by Green and Swets (1966). In a typical experiment from that time, the researcher presented auditory stimuli containing either noise or noise plus a faint tone to participants. They, in turn, had to indicate whether they perceived a tone embedded in the noise or not. Therefore, even today, the two categories of stimuli are generally called “noise” and “signal”. Since the days of the first experiments, SDT has found numerous applications in many fields, such as diagnostics, quality control and psychology.

Although every psychology student comes across topics, questions and studies which should call for the application of SDT, the theory and its applications are not widespread and mostly not compulsory content in psychology curriculum. Instead, numerous studies can be found in which a research question from the field of SDT is examined, but the analysis of the data remains on the very basic descriptive level of counting and comparing correct and wrong answers (for a discussion of this problem, see Stanislaw & Todorov, 1999, p. 137). Hence, precious opportunities of a comprehensive data analysis are wasted, and conclusions which would be possible due to some additional analyses cannot be drawn. Furthermore, even if researchers analyse data with SDT, they rarely conduct either an a priori analysis to determine the necessary sample size or an a posteriori analysis to calculate the statistical power.

As far as we can see, there are only a few non-specialist, step-by-step introductions which offer guidelines for the application of SDT to empirical research (e.g., Macmillan & Creelman, 2005; Schiffman, 2005; Sorkin, 1999; Stanislaw & Todorov, 1999; Treat & Viken, 2012; Wickens, 2002). However, these sources are not comprehensive, as they do not include the A–Not A design, relevant significance tests against chance level, or considerations regarding statistical power and sample size. Such information can only be found in statistical publications (e.g., Bi, 2015; Ennis & Jesionka, 2011). However, these publications are hard to understand for researchers from other fields of psychology and contain little guidance for experiment planning and conducting. Surprisingly, guidelines such as *The Reviewer's Guide to Quantitative Methods in the Social Sciences* (Hancock et al., 2019) and the *Journal Article Reporting Standards* from the American Psychological Association (2019) do not present any designs from the family of SDT.

Therefore, this paper tries to bridge the gap between theoretical knowledge in formal mathematical publications and the researcher who—in general—does not have much knowledge about the topic but is interested in the development of study designs in line with SDT. We start with the description of forced-choice and A–Not A methods and then focus on the A–Not A (also designated as *yes–no*) design, explaining descriptive and inference statistical procedures. This design is used not only in the domain of music psychology but also in other psychological disciplines (e.g., Al et al., 2020; Cameron et al., 2004; Clemens et al., 2015; Sorkin et al., 2001; Tsoi et al., 2008; Viswanathan et al., 2017; Wyart et al., 2012). Study designers using an A–Not A task present only one stimulus to participants at a time. They, in turn, classify the stimulus as belonging to one of two categories. The A–Not A design can be divided into several sub-designs, and this paper will focus on the non-replicated as well as the replicated paired A–Not A task. Paired tasks should be used whenever the set of stimuli can be grouped into pairs by content-related qualities. For example, the stimulus set might consist of different musical pieces presented in two conditions each or of different sentences provided in two slightly different wordings each.

Sample data files, Excel files and R scripts are given for the reader to retrace the analysis procedure. The main contribution of this paper is the section of sample size and power calculation: To the best of our knowledge, this is the first publication to comprehensively explain aspects of statistical power and sample size, give supplementary material for practicing and conducting a study employing the paired A–Not A design, and propose a best-practice procedure for conducting such a study.

After calculating an effect size, researchers usually apply benchmarks to the calculated value to classify the effect as small, medium, or large (Ellis, 2010). For the SDT-specific effect size *d′*, benchmarks are hard to find and need to vary depending on the specific design employed. This paper summarizes the available information on benchmarks and points to theoretical inconsistencies. These issues should be addressed by future research on the benchmarks for *d′* in various SDT designs.

## Signal detection theory and its different designs

### Examples of study designs from the signal detection family

As mentioned in the introduction, SDT is applicable on a wide range of topics and in many scientific disciplines. We collected studies employing SDT which are listed in Table S1 (see Supplementary Materials available from https://osf.io/tvsj5/?view_only=28c54b315737438faaea3837f92528b9, “1 Studies Using SDT Designs.pdf”) along with some details about the research designs employed in the studies which come from the broad field of auditory and visual perception research, as well as other psychological disciplines. These studies offer a convenience sample of research employing SDT methods and were not obtained by systematic review. Furthermore, there are several publications highlighting the relevance of SDT for specific fields of research, such as social psychology (Martin & Rovira, 1981), sales effectiveness (Knowles et al., 1994), food sensory science (O’Mahony & Hautus, 2008), or advertising recognition (Tashchian et al., 1988).

One very recent study is used throughout this paper as an example, and its data are provided as sample data in the Supplementary Material. Düvel et al. (2020) conducted a study comparing the sound of original hardware guitar amplifiers with simulations of this specific sound by the Kemper Profiling Amp. The stimuli consisted of pairs with the same musical excerpt performed by the same guitarist recorded either from the original amp (OA, Type A as defined in the section “The paired A–Not A design: Data structure and analysis” of this paper) or the Kemper Profiling Amp (KPA, Type Not A as defined in the same section). Response data were based on six pairs of stimuli (i.e., six musical examples each produced in two different ways, one way using the OA and the other the KPA, resulting in 12 stimuli) rated by 177 participants. The study by Düvel et al. (2020) was chosen as the sample data set as it employed the exact kind of research design which is the focus of the present paper. Furthermore, the research question is relevant for current discourse in popular music research and easy to understand for non-experts.

### Forced-choice and A–Not A designs

Although those who use SDT as a framework always aim to quantify a discrimination performance, they can choose from a variety of designs and methods. These designs can be classified in different ways: one is proposed by Bi (2015, S. 5–7), although this may not be the most common classification in the field psychology. In the category of forced-choice tasks, two or more stimuli are always presented at the same time. The *two-alternative forced-choice* design (2-AFC design) is probably the most common one from this category. Here, two stimuli, one of stimulus type A and one of B, are presented in one trial, and participants have to indicate which of the two belongs to which category (A or B). In contrast, in an A–Not A method, only one stimulus (of type A or Not A) is presented at a time and is classified as belonging to one of the two categories (A and Not A). These two methods show different kinds of response bias. Forced-choice methods can show *position bias* (by García-Pérez & Alcalá-Quintana, 2011, labelled as *interval bias*), whereas A–Not A methods show *criterion bias* (Kroll et al., 2002, p. 243). In an A–Not A design, participants might answer A more often than B or vice versa. This is especially prevalent in difficult tasks in which participants make uncertain judgements (Bi, 2015, p. 6). The quantification of this response bias, called criterion bias, is achieved by calculating the measure *c* (see section “Effect sizes for the discrimination ability” in this paper). In forced-choice methods, both types of stimuli always have to be presented during one trial. Moreover, participants have to be informed that both sides are always presented. The occurrence of the other type of response bias, called position bias, can be illustrated with a two-alternative forced-choice (2-AFC) task. Participants are presented with two stimuli at the same time (one of Type A, one of Type B) and have to indicate which one of them is A and which one B. As explained by Jou et al. (2016, p. 33), by design, participants cannot choose Option A more often than Option B. Therefore, if the measure *c* is calculated based on all items, it would result in no bias (and be 0). Only when calculating *c* with either the left or the right response options, does it differ from 0 (Jou et al., 2016, p. 33). Of course, we can also consider the pair of stimuli as a unit: Participants can classify the two stimuli more often as A and B and not B and A, for example. This would result in a response bias called position bias.

### Distinction of different A–Not A designs

A–Not A designs include designs in which participants are presented with one stimulus at a time and have to allocate it to one of two different answer categories (A or Not A which is equal to Signal or Noise). These designs are also called *one-interval* designs (Macmillan & Creelman, 2005, p. 1) or yes–no tasks (Green & Swets, 1966, pp. 32–35; Wickens, 2002, pp. 4–5). In terms of the number of participants and stimuli, several sub-designs are possible and classified in Table 1.

**Table 1:** Classification of A–Not A sub-designs (Bi & Ennis, 2001a, p. 216; 2001b, p. 344)

	Monadic	Mixed	Paired
Non-replicated	Each participant evaluates only one stimulus (A or Not A).	Each participant draws a random stimulus set from the stimulus pool. The number of possible stimuli should be much larger than the number of participants.	Each participant evaluates only one pair of stimuli (a pair consisting of one A and one Not A).
Replicated	Each participant evaluates more than one stimulus of either A or Not A but not both.	Each participant evaluates more than one stimulus of A and Not A.	Each participant evaluates more than one pair of A and Not A stimuli (same number of A and Not A stimuli).

For the distinction between *monadic*, *mixed*, and *paired* designs, see Bi (2015, pp. 70–77). If participants are presented with just one stimulus or one pair of stimuli, the designs are non-replicated. If this procedure is repeated, the design is called replicated. The number of replications indicates the number of successively presented pairs of stimuli (though mostly in randomized order). For non-replicated designs, considerations concerning statistical testing and the relation between sample size and test power are given in Bi (2015, pp. 70–87). For replicated designs, additional information is given in Bi (2015, pp. 301–328). Due to its application in the field of music perception research, the paired design is our focus in this paper. Subsequently, two stimuli, one of stimulus type A and one of type Not A, always form a pair due to their content. For example, it can be the same musical piece performed in two conditions (A and Not A; as in Düvel et al., 2020), the same statement varying in its wording (where wording A is compared to wording Not A), or the same motive in two pictures varying in a particular way (which manifests in stimulus type A and type not A). In a replicated task, several pairs of, for example, musical performances, statements, or pictures would be presented in one study in randomized order.

In the following sections, the paired design is explained in more detail, and considerations concerning statistical power and required sample size are made for non-replicated as well as replicated paired designs.

### Comparing the replicated paired A–Not A and the 2-AFC design, their applications and ecological validity

The A–Not A and the 2-AFC designs are sometimes confused (see, for example, Kopiez et al., 2016). In the A–Not A design, two alternative response options are also given (“The stimulus belongs to stimulus class A” and “… to stimulus class Not A”), and the participant is forced to select one of these options. Therefore, it seems obvious to name this a two-alternative forced-choice (2-AFC) design—but this decision is incorrect. The correct label of this procedure would be A–Not A design (or yes–no, sometimes also called one-interval design). As described in the previous section, in a 2-AFC experiment, two stimuli are presented at the same time, one belonging to Stimulus Class A and one to Not–A; the participant has to decide the correct classification.

The A–Not A design and the 2-AFC design measure different kinds of response bias. In the 2-AFC design, the participant is aware that the two stimuli always belong to the two different categories. Therefore, they either allocate both correctly or both incorrectly and cannot tend (i.e., in the sense of a criterion bias) toward one response category more often than to the other. However, in some contexts, it might be of interest to measure participants’ criterion bias. In these cases, researchers must take care when selecting a research design for their study and select one which is able to measure the desired kind of bias.

Additionally, researchers should consider the ecological validities of different designs regarding their research question: the degree to which the situation under study resembles the real-world situation. Whereas classifying a stimulus in practice normally does not involve comparing the two possible conditions, the A–Not A has a higher ecological validity compared to the 2-AFC design. For example, people normally listen to just one performance of music when trying to decide about the presence or absence of a particular feature. In different perceptual modalities (taste, vision, hearing, tactile sense, …), the comparison of several stimuli (as in 2-AFC) might be more prevalent than the classification of a singular stimulus (as in A–Not A). Therefore, the former might yield higher ecological validity in these cases.

In the following sections, these considerations will be illustrated by a study by Düvel et al. (2020) on the identification of sounds recorded from original amplifiers and simulations made with the Kemper Profiling Amp. In practice, a person might listen to some music and wonder whether the guitar sound was produced by an original amplifier or by a digital simulation. In this case, the listener does not have the possibility of direct comparison, hearing the same piece of music under both recording conditions. Therefore, it seems reasonable to decide for a similar design in the empirical study and to present only one stimulus at a time (using the A–Not A design).

On the other hand, the 2-AFC design yields minor advantages over the A–Not A design concerning statistical power (Bi, 2015, p. 6; Bi & Ennis, 2001b, pp. 354–357). Slightly more participants are needed for an A–Not A study than for a 2-AFC study to detect the same effect size under the condition of same α- and β-error thresholds (α- and β-errors [also called type I and type II errors] are explained in the section “Mathematics for the calculation of test power and sample size” in this article).

## The paired A–Not A design: Data structure and analysis

### Descriptive statistics of responses

In a paired A–Not A design, participants evaluate one at a time two stimuli which represent a matched pair due to their content-related qualities of interest. The two stimulus classes are called A and Not A, and participants have to allocate each of the given stimuli to one of the two possible response categories. Therefore, it is possible to allocate both stimuli of one pair to the correct categories (called pattern *c*), both to the incorrect categories (the A stimulus to stimulus class Not A and vice versa, called pattern *b*), or to allocate one correctly and one incorrectly. The latter results in two possibilities: Participants either allocate the A stimulus to the correct stimulus class and the Not A stimulus to the incorrect stimulus (therefore, answer A two times, pattern *a*), or they allocate the A stimulus to the Not A stimulus class and the Not A stimulus to the same (here correct) stimulus class (answer Not A two times, pattern *d*). These possible answer patterns are shown in Table 2. This statistical information can also be displayed as a tree diagram (see Figure S1 in the Supplementary Material, file “1 Studies Using SDT Designs.pdf”). The tree diagram contains the same information as Table 2 and may serve as complementary information for understanding the table.

**Table 2:** Classification of response patterns in a paired A–Not A design

		Stimulus is A		Sums of columns
	Participant says …	“A”	“Not A”	
Stimulus is Not A	“A”	*a*	*b*	*a*+*b* = number of FAs
	“Not A”	*c*	*d*	*c*+*d* = number of CRs
Sums of rows		*a*+*c* = number of Hits	*b*+*d* = number of Misses	*a*+*b*+*c*+*d* = *N*

The table displays frequencies of response pattern to stimuli pairs. FAs = false alarms, CRs = correct rejections, and *N* = effective sample size

The classification of the participants’ responses in response patterns might be new for most readers. The more prevalent way of classifying responses from SDT tasks is the calculation of hit, miss, false alarm, and correct response rates based on the responses to each stimulus individually. This classification is used for the calculation of the sensitivity *d′*, as explained in the section “Effect sizes for the discrimination ability”. However, for the corresponding significance test as well as considerations concerning test power and sample size (see the following sections), it is necessary to consider the structure of pairs in the data and instead use pairs of responses for the calculations.

Table 2 records the frequency of the response patterns to a pair of stimuli. If a non-replicated design is applied, each participant evaluates just one pair of stimuli, and the response is classified as either *a*, *b*, *c*, or *d*. In the case of a replicated design, each participant evaluates *k* pairs of stimuli and, therefore, creates *k* response patterns for each of the *k* stimulus pairs. In this case, the number of participants *n* does not equal the effective sample size *N* in Table 2, but the effective sample size *N* is *k* times the number of participants *n*, N = k*n. In the rest of this article, we always discriminate between the number of participants *n* and the effective sample size *N*, which is the number of responses to stimulus pairs.

For the analysis of data, it is crucial to count the answer patterns to the pairs of stimuli. Two ways of conducting these calculations in Excel and R are explained in the section “In practice: Calculating the descriptive statistics and effect sizes”.

### Significance test for the response behaviour

The next question is, does the behaviour of the participants differ between A and Not A stimuli? If the response behaviour is the same, participants decide on chance level and have no ability to discriminate between the two types of stimuli. Our null hypothesis would, therefore, be that they answer A equally often for A and for Not A stimuli. The probability of giving response A to stimulus A ($={p}_A=\frac{a+c}{N}$) would be the same as the probability of giving response A to stimulus Not A ($={p}_N=\frac{a+b}{N}$, *H*_0_ : *p*_*A*_ = *p*_*N*_ ). The alternative hypothesis would be that the probability of giving the correct answer A to a stimulus A differs from the allocation of stimulus Not A to A (*H*_1_ : *p*_*A*_ ≠ *p*_*N*_).

These hypotheses can be tested using McNemar’s test, as both the dependent and the independent variable have two categories (A and Not A), and the data are matched (one participant creates two answers; thus, the answers are not independent; McNemar, 1947). To compare *p*_*A*_ and *p*_*N*_, we just have to consider *b* and *c*, as the other variables *a* and *N* are included in both *p*_*A*_ and *p*_*N*_ (${p}_A\overset{?}{=}{p}_N\iff \frac{a+c}{N}\overset{?}{=}\frac{a+b}{N}\iff a+c\overset{?}{=}a+b\iff c\overset{?}{=}b$). The test statistic for McNemar’s test without a continuity correction is given in Eq. 1 and follows an asymptotic chi-square distribution (*df* = 1).

1 $${X}_{McNemar}^2=\frac{{\left(b-c\right)}^2}{b+c}$$

For the calculation of McNemar’s test, the frequencies *b* and *c* from Table 2 are required (for information on the calculation, see the section “In practice: Calculating the descriptive statistics and effect sizes”).

There are several possibilities for continuity corrections (Bi, 2015, pp. 76–77; Bi & Ennis, 2001a, p. 222; Fay, 2010, p. 55). Nowadays, it is also possible to calculate the exact test using a binomial distribution, and this method should be preferred to the approximate McNemar’s test (with or without continuity correction). If the ${X}_{McNemar}^2$ value exceeds the critical value for the given significance level (always one degree of freedom; the critical value for *p* ≤ .05 is 3.84), we can reject the null hypothesis and accept the alternative hypothesis that participants allocate stimuli A with a different probability to stimulus class A than stimuli Not A to stimulus class A. Actually, we are not only interested in this two-tailed hypothesis but also want to rule out that participants show reversed allocation behaviour by allocating stimuli A to stimulus class Not A and vice versa above chance level. This could be due to the participants either deliberately giving wrong answers or their having understood the properties of the stimulus classes or the task in an interchanged way. Therefore, the one-tailed alternative hypothesis would be preferred to the two-tailed and would be that participants allocate stimuli of stimulus class A more often to response category A than to response category Not A. The null hypothesis then is that participants allocate stimulus A more often to stimulus class Not A or to both categories equally often.

If we want to test a one-tailed hypothesis, we can use half of the usual significance level provided that the frequency of response pattern *c* indeed exceeds the frequency of response pattern *b*. For *p* ≤ .025, the critical value of the ${X}_{McNemar}^2$ distribution is 5.02. If the test value exceeds this threshold, we can assume that participants allocate stimuli A more often to stimulus class A than stimuli Not A to stimulus class A.

To avoid a manual calculation of the test statistic using Eq. 1, the R script “3 Calculating McNemar's Test.R” from the Supplementary Material can be used. We recommend opening the R scripts from this paper in RStudio. The first section in the script works exactly as it does in Eq. 1. The second section gives the code for the calculation of an exact McNemar’s test using the binomial distribution. This function gives not only the *p*-value for the calculation but also a 95% confidence interval and a sample estimate for the effect size odds ratio (see next section).

Further information on McNemar’s test, its continuity corrections, and the exact version of the test was summed up by the author of the R package “exact2x2” (Fay, 2016).

### Effect sizes for the discrimination ability

The effect size *w* (also called phi) for this discrimination ability can be easily determined after calculating McNemar’s test using Eq. 2 (Bi, 2015, p. 76).

2 $$w=\sqrt{\frac{X_{McNemar}^2}{N}}$$

The benchmarks for the effect size *w* are: small if .1 ≤ *w* < .3, medium if .3 ≤ *w* < .5, and large if *w* ≥ .5 (Ellis, 2010, p. 41). This effect size *w* can also be calculated by the R script “3 Calculating McNemar's Test.R” from the Supplementary Material (see its Section 3). However, *w* was shown to have poor statistical qualities and depends heavily on the marginal frequencies (*a+b*, *c+d*, *a+c*, and *b+d* in Table 2) of the contingency table (see Olivier & Bell, 2013, for details).

Another effect size for McNemar’s test is the odds ratio *OR*, which is calculated as

3 $$\mathrm{O}R=\frac{b}{c}$$

(Bi, 2015, p. 78; Faul et al., 2007, p. 187) with frequencies *b* and *c* from Table 2. By Olivier et al. (2016), this *OR* is referred to as Mantel–Haenszel odds ratio, and the following are benchmarks for the effect size: small if 1.22 ≤ *OR* < 1.86, medium if 1.86 ≤ *OR* < 3.0, and large if *OR* ≥ 3.0.

Within the framework of SDT, effect sizes for the quantification of the discrimination or allocation ability and bias of participants were developed. After calculating McNemar’s test, these SDT-specific effect size *d′* (“d prime”) and *c* for the answering bias can be calculated. However, in the case of a non-significant McNemar’s test (meaning that the participants’ allocation ability was no better than chance level), the user should be cautious with the interpretation of these measurements. For *d′*, the quantiles for the standard normal distribution of the proportions of hits and false alarms are determined (*z*[*p*_*Hits*_]*,* and z[*p*_*FAs*_]), and their difference is calculated (Bi, 2015, p. 12; Wickens, 2002, p. 24):

4 $$d^{\prime }=z\left({p}_{Hits}\right)-z\left({p}_{FAs}\right)$$

For the calculation of *d′*, homoscedasticity is assumed, that is, both distributions of responses to stimulus classes A as well as Not A along the participants’ decision space are expected to show equal variances (Bi, 2015, p. 46; Hautus, 2015, p. 947; see Macmillan & Creelman, 2005, p. 17 for a sample figure of these distributions). For analysing data which show unequal variances, see Hautus (2015, p. 948) and Macmillan and Creelman (2005).

As *p* = 0 or 1 for *p*_*Hits*_ and *p*_*FAs*_ would lead to *z(p)* = −∞ or ∞, these proportions have to be corrected before the calculation of the quantiles. Several ways of correcting values of *p* = 0 or 1 are discussed by Miller (1996, pp. 66–67) and (Sorkin, 1999, pp. 51–52). Another method is suggested by Macmillan and Creelman (2005, p. 8), who substitute 0 by 1/(2*k) and 1 by 1−1/(2*k), with *k* being the number of stimulus pairs. This correction has proven practical in previous studies as the absolute values of the resulting *z* values are still large but do not deviate strongly from the distribution of the other *z* values. We have used this approach in this paper.

This effect size can be calculated for most designs within SDT but should not be compared between studies based on different designs. Benchmarks for *d′* for the A–Not A design can be found in Bi (2015, p. 44): A rather small effect is 0.0 < *d′* < 0.74, a meaningful effect is 0.74 < *d′* < 1.81, and a rather large effect is *d′* > 1.81. The magnitude of *d′* in a 2-AFC task differs and can be expected to be about 41% larger (factor $\sqrt{2}$) than in an A–Not A design ($d^{\prime}_{2- AFC}=\sqrt{2}\ast d^{\prime}_{A- NotA}$; Green & Swets, 1966, p. 68; Wickens, 2002, p. 104). Benchmarks for *d′* in a 2-AFC design can be found in Ennis and Jesionka (2011, p. 380): A small effect is 0.5 ≤ *d′* < 1.0, a medium effect is 1.0 ≤ *d′* < 1.5, and a large effect is *d′* ≥ 1.5. These two sets of thresholds for the classification of effect sizes and the information on factor $\sqrt{2}$ do not seem to stand in a relationship of linear transformation (with factor $\sqrt{2}$) to each other. The summary and comparison of benchmarks of *d′* for different designs within the SDT family remain a subject of future research.

The section “Overview of designs” explains the difference between A–Not A designs, which can reveal a criterion bias, and forced-choice designs, which can show the second form of response bias, the so-called position bias. For designs with criterion bias, it is recommended to calculate one of several measures of this bias to quantify a participant’s answering tendency (Macmillan & Creelman, 2005, pp. 28–41). The most common measure for criterion bias is *c*:

5 $$c=-\frac{1}{2}\ast \left[z\left({p}_{Hits}\right)+z\left({p}_{FAs}\right)\right]$$

Here, *c* = 0 indicates a balanced selection of both response pattern, and *c* > 0 indicates a tendency to say “No” (i.e., the miss rate is larger than the false alarm rate), whereas *c* > 0 indicates a tendency to say “Yes” (i.e., the false alarm rate is larger than the miss rate; Macmillan & Creelman, 2005, p. 29). This response bias index *c* is not the same as the frequency *c* in Table 2; the two measures just accidently bear the same name.

### In practice: Calculating the descriptive statistics and effect sizes

In the following scenario, data have been acquired in a paired A–Not A study. Each row contains the data of one participant, and each column displays the results for a certain stimulus. In the case of a non-replicated paired design, there will be just two data columns with one column for each of the two stimuli. In the case of *k* replications (= number of stimuli pairs), the data set consists of 2*k columns. The stimuli are numbered so that Stimuli 1 and 2, 3 and 4, etc. represent a pair; the odd-numbered stimuli (1, 3, 5, …) belong to Type A, and the even-numbered (2, 4, 6, …) to Type Not A. For the analysis in Excel, it is easiest to sort the variables according to the types A and Not A. The cells contain the responses from the participants to the respective stimulus. The coding of the response A is 1 and Not A is 0. See Table 3 for an example. Here we can see that Participant 1 made two mistakes for the A stimuli by estimating Stimuli 1 and 2 as being Not A and by allocating the Not A stimuli altogether incorrectly.

**Table 3:** Example data from a replicated paired A–Not A study with *n* participants and *k* = 3 replications. Response “A” is coded as 1 and response “Not A” as 0

Participant	Stim01_A	Stim03_A	Stim05_A	Stim02_NotA	Stim04_NotA	Stim06_NotA
1	0	0	1	1	1	1
2	0	1	0	0	0	1
…	…	…	…	…	…	…
n	1	0	1	0	0	0

For the calculation of the frequencies *a*, *b*, *c*, and *d*, and the effect sizes sensitivity *d′* and bias *c*, there are two possibilities offered in the Supplementary Material: Either the Excel sheet or the R script can be used. Both yield the same results. For this example, both files use data from the study by Düvel et al. (2020) described earlier.

#### Calculating in Excel

The first spreadsheet “n and k” within the Excel document from the Supplementary Material (“4 Calculating the Descriptive Statistics and Effect Sizes.xlsx”) contains two pieces of information: the number of participants *n* and the number of replications *k*. Users should change the two values here (grey cells) according to their own data. Column A from the second spreadsheet “Data and Calculations” contains the number of each participant and columns B to M contain their responses to the (in this case) 12 stimuli. If more or fewer than six replications are used, the number and names of the columns have to be adjusted accordingly. Also, the number of rows has to be adjusted to the number of participants and the formulas copied by dragging down the fill handles in the columns N to AE if participants have been added. When entering data in columns B to M (grey cells), the following columns (N and higher) are calculated by the internal formulas. These columns calculate the frequencies *a*, *b*, *c*, and *d*, the number of hits, false alarms, misses, correct rejections (all as from Table 2), answers A, and answers Not A, the proportions of hits and false alarms, their corrections for the following *z*-transformation (as described in the section “Effect sizes for the discrimination ability”), and the calculated sensitivity *d′* (as in Eq. 4) as well as the response bias *c* (as in Eq. 5) for each participant from the data. The third spreadsheet, “Outcomes”, is filled in automatically, but it should be checked whether the formulas correspond to the correct cells in the second spreadsheet (e.g., if rows have been added, the numbers have to be adjusted). The “Outcomes” spreadsheet contains a summary of the data: the number and proportion of correct answers for each stimulus; the frequency and proportion of the response patterns *a*, *b*, *c*, and *d*; frequencies and proportions of hits, false alarms, misses, and correct rejections; and mean, standard deviation, minimum, and maximum of the proportions of hits and false alarms, their corrected variables, their *z*-transformations and the resulting sensitivity *d′* and bias *c.*

#### Calculating in R

For the calculation of the same descriptive measures and effect sizes in R, the data have to be provided in a CSV file to be loaded into R. The corresponding file from the Supplementary Material is labelled “5 Data for the Calculation of the Descriptive Statistics and Effect Sizes in R.csv” (see Supplementary Material) and contains the data from Düvel et al. (2020) exactly as the Excel file from the section *Calculating in Excel*. If you have collected your own data, enter them into the document and adjust the column names and numbers of rows if necessary. Then open the R script “6 Calculating the Descriptive Statistics and Effect Sizes.R”. First, the working directory has to be defined by changing the path in the first line. Then, the CSV data file from your working directory can be loaded (the file has to be in that folder).

In the following Section 1 of the R script, first, enter the number of replications *k* and the number of participants *n* (replace 6 and 177 with the numbers from your study). In a next step, the variables are calculated analogous to the calculation in columns N to AE in the Excel document. The code for the calculation of *a*, *b*, *c*, *d*, hits, and false alarms has to be adjusted if you used more or less than six replications. The patterns should be easily recognizable, and comments after the number sign (#) at the end of some lines lead to the points where the code might need adjustment. Afterwards, all the lines in Section 1 have to be executed by using *Command* and *Enter* (Mac) or *Ctrl* and *Enter* (Windows & Linux). You can either place the curser in the first line, execute it and proceed with all the next lines, or you select the whole section and execute it using the keyboard shortcut. The last line in Section 1 creates a new CSV document in your working directory with the name “7 Calculating the Descriptive Statistics and Effect Sizes_Results from R.csv”. This output file contains all the newly calculated variables. The second section of the R script calculates the descriptive analysis of the data and produces histograms of the sensitivity indicator *d′* and bias *c*.

### The questionable use of a one-sample *t*-test for testing against chance level

Many researchers regard the calculated sensitivity *d′* as a dependent variable and treat it accordingly. We came across some studies in which researchers calculated the sensitivity for each participant followed by a test against chance level (*d′* = 0) to determine whether the participants’ discrimination abilities differed significantly from chance level. In other studies, researchers used an ANOVA to test different groups for significantly different detection abilities (for examples of these procedures, see Bartlett et al., 1995; Bergeson & Trehub, 2006; Kopiez et al., 2016; Schellenberg & Trehub, 1996; Trainor & Trehub, 1993; Trehub et al., 1990; Trehub & Hannon, 2009).

However, as revealed by Bi (2015), the adequate procedure to determine whether participants’ response behaviour differs significantly from chance level is the McNemar’s test (see the section “Significance test for the response behaviour”). It is applied to the most basic level of the data, namely, the frequencies and proportions of the response patterns. Therefore, the widely distributed procedure of first calculating the effect size *d′*, treating it as a dependent variable and then using a *t*-test to test for significance seems more like a detour and an imprecise procedure.

Therefore, we suggest using McNemar’s test to test for significant response behaviour instead of using a one-sample *t*-test on the *d′* values.

## The statistical power of the (non-replicated) paired A–Not A design

Whereas some publications present considerations concerning the test power for the 2-AFC and some other forced-choice designs (Ennis & Jesionka, 2011), the publications by Bi (2006, 2015) provide considerations about test power and sample size for forced-choice designs (Bi, 2015, pp. 65–70) as well as A–Not A designs. In this section, we will focus on the test power of the non-replicated and replicated paired A–Not A design.

Approximations for the calculation of statistical power and the required sample size for a paired A–Not A design are presented in Bi (2015, pp. 84–87). The corresponding R scripts are explained in the section “Practical procedure for the calculation of power and sample size using R”. Furthermore, the software G*Power provides approximated and exact calculations of the power for the McNemar’s test (Faul et al., 2009). This procedure will be described in the section “Practical procedures for the calculations of power and sample size using G*Power”.

### Mathematics for the calculation of test power and sample size

Let us assume that each participant evaluates only one pair of stimuli (which equals a non-replicated design) and, therefore, the sample size (being the number of response pairs) equals the number of participants. The section “Adjustment from a non-replicated to a replicated paired A–Not A design” of this article will differentiate between replicated and non-replicated designs.

Several approaches to the question of the required sample size in this design can be found in the literature. They result in slightly different numbers, and one cannot easily declare one as right and the others as wrong. Therefore, several approaches will be presented in this paper, and results will be compared. We leave it up to the reader and researcher to choose the appropriate procedure. Basically, the thresholds for α-error (also called type I error), β-error (also called type II error; with the resulting test power 1−β), and the expected effect size for a specific statistical test determine the required sample size. As an implicit convention, the following standards have been widely accepted: The α-error (i.e., the probability of the test finding a difference between groups when there actually is no difference) is set to 5%, which results in the usual significance level of *p* < .05. Furthermore, the β-error (i.e. the probability of overlooking a difference between groups when it is actually there) is set at 20%. This results in a statistical power of at least 80% (since power = 1− β). According to Cohen (1988, p. 56), this adjustment is called the “.20/.05” convention.

As described in the previous sections, our aim is to calculate McNemar’s test to determine whether participants can allocate stimuli above chance level. To calculate the required sample size for the power analysis, a priori information on the size of the difference between groups is required, which comes down to the frequencies *b* and *c* (see Table 2) and their proportions of the effective sample size *N*, *p*_*b*_ (= *b/N*) and *p*_*c*_ (= *c/N*).

Two different approaches for the approximate calculation of test power are presented: the first by Miettinen (1968), the second by Bennett and Underwood (1970). According to Miettinen (1968), the statistical power is calculated as follows:

6 $$Powe{r}_M=\varPhi \left(\frac{-{z}_{1-\alpha}\psi +\sqrt{N\psi}\left(2\varDelta \right)}{\sqrt{\psi^2-4{\varDelta}^2}}\right)$$

where *Φ* is the distribution function of the standard normal distribution, α is the significance level (i.e., the acceptable proportion of an α-error), *z*_1−α_ is the 100(1−α)-percentile of the standard normal distribution, 2*Δ* = *p*_*c*_ − *p*_*b*_ (therefore, $\varDelta =\frac{p_c-{p}_b}{2}$), *ψ* = *p*_*c*_ + *p*_*b*_ and *N* is the sample size. Accordingly, the required sample size *N* for a one-tailed McNemar’s test can be calculated by

7 $$N=\frac{{\left[{z}_{1-\alpha}\psi +{z}_{1-\beta}\sqrt{\psi^2-{\left(2\varDelta \right)}^2}\right]}^2}{\psi {\left(2\varDelta \right)}^2}$$

The same approach is described by Machin et al. (2009, p. 70) although slight differences in variable naming might confuse the reader at first sight. This approach is also employed in the software PASS (power analysis and sample size software, described by NCSS, n.d.).

For the Bennett and Underwood approach (1970), *p* = *p*_*b*_ + Δ = *p*_*c*_ − Δ, $g=\varDelta \sqrt{N}$ and $\lambda =\frac{2{g}^2}{p}$ are additionally needed. The statistical power for the one-tailed test can be calculated as

8 $$Powe{r}_{BU}=\Pr \left({X}_1^2\left(\lambda \right)>{k}_{1-\alpha}\right)$$

which is the probability of ${X}_1^2\left(\lambda \right)$ (= a noncentral chi-square distribution with one degree of freedom and noncentral parameter 𝜆) being larger than *k*_1−α_ (= critical value of a chi-square distribution with one degree of freedom and significance level 𝛼 in a one-tailed test). This equation cannot be disintegrated for the sample size, but solutions have to be calculated numerically. Some solutions are given in Tables 4.10 and 4.11 in Bi (2015, p. 86), and more combinations can easily be calculated by the R script explained in the following section Table 5.

**Table 4:** Comparison of calculated required sample sizes by different calculation methods with α-error probability = .05 and power = .8. Methods are 1. G*Power, Option Faster Approximation; 2. G*Power, Option Exact; 3. R Script: mcn_bu_N(pb, pc), 4. R script: mcn_m_N(pb, pc). Each cell contains the corresponding *p*_*b*_ = *b/N* and *p*_c_ = *c/N* for the given odds ratio (OR) and proportion of discordant pairs (*p*_*D*_) as well as the required sample sizes according to methods 1 to 4

	Odds ratio: *OR* = 5	Odds ratio: *OR* = 4	Odds ratio: *OR* = 3	Odds ratio: *OR* = 2	Odds ratio: *OR* = 1.5
Proportion of discordant pairs: *p*_D_ = .2	*p*_b_ = .1667 *p*_c_ = .0333 1. 65 2. 75 3. 70 4. 59	*p*_b_ = .16 *p*_c_ = .04 1. 90 2. 92 3. 86 4. 75	*p*_b_ = .15 *p*_c_ = .05 1. 115 2. 134 3. 124 4. 113	*p*_b_ = .1333 *p*_c_ = .0667 1. 290 2. 299 3. 279 4. 269	*p*_b_ = .12 *p*_c_ = .08 1. 790 2. 814 3. 773 4. 763
Proportion of discordant pairs: *p*_D_ = .3	*p*_b_ = .25 *p*_c_ = .05 1. 44 2. 50 3. 47 4. 39	*p*_b_ = .24 *p*_c_ = .06 1. 60 2. 61 3. 58 4. 50	*p*_b_ = .225 *p*_c_ = .075 1. 77 2. 89 3. 83 4. 76	*p*_b_ = .2 *p*_c_ = .1 1. 194 2. 199 3. 186 4. 179	*p*_b_ = .18 *p*_c_ = .12 1. 527 2. 543 3. 516 4. 509
Proportion of discordant pairs: *p*_D_ = .4	*p*_b_ = .3333 *p*_c_ = .0667 1. 33 2. 37 3. 35 4. 30	*p*_b_ = .32 *p*_c_ = .08 1. 45 2. 46 3. 43 4. 38	*p*_b_ = .3 *p*_c_ = .1 1. 58 2. 67 3. 62 4. 57	*p*_b_ = .2667 *p*_c_ = .1333 1. 145 2. 149 3. 139 4. 134	*p*_b_ = .24 *p*_c_ = .16 1. 395 2. 407 3. 387 4. 382
Proportion of discordant pairs: *p*_D_ = .5	*p*_b_ = .4167 *p*_c_ = .0833 1. 26 2. 30 3. 28 4. 24	*p*_b_ = .4 *p*_c_ = .1 1. 36 2. 37 3. 35 4. 30	*p*_b_ = .375 *p*_c_ = .125 1. 46 2. 53 3. 50 4. 46	*p*_b_ = .3333 *p*_c_ = .1667 1. 116 2. 119 3. 112 4. 108	*p*_b_ = .3 *p*_c_ = .2 1. 316 2. 326 3. 310 4. 305
Proportion of discordant pairs: *p*_D_ = .6	*p*_b_ = .5 *p*_c_ = .1 1. 22 2. 25 3. 24 4. 20	*p*_b_ = .48 *p*_c_ = .12 1. 30 2. 30 3. 29 4. 25	*p*_b_ = .45 *p*_c_ = .15 1. 39 2. 44 3. 42 4. 38	*p*_b_ = .4 *p*_c_ = .2 1. 97 2. 99 3. 93 4. 90	*p*_b_ = .36 *p*_c_ = .24 1. 264 2. 271 3. 258 4. 255

**Table 5:** Data structure for the calculation of the adjustment factor *C.*

	Answers “A”	Answers “Not A”
Participant 1	5	7
Participant 2	6	6
Participant 3	6	6
…	…	…
Participant *n*	5	7

The first column is displayed here only for explanation. In this example, the number of replications *k* is 6; therefore, the sum of each row is always 12 (responses to 6 pairs of stimuli)

### Practical procedure for the calculation of power and sample size using R

The R-script “8 Calculations of Power and Sample Size.R” in the Supplementary Material calculates the power and sample size according to the previous section. Four functions are defined in the script: “mcn_m_p”, “mcn_m_N”, “mcn_bu_p”, and “mcn_bu_N” with “mcn” standing for McNemar’s test, “m” for Miettinen’s approach, “bu” for Bennett and Underwood’s approach, “p” for power, and “N” for sample size. Therefore, these functions relate to Eqs. 6, 7, 8, and the numerical reversal of Eq. 8. After opening the document in R (or preferably in RStudio), the code of the desired function has to be executed using *Command* and *Enter* (Mac) or *Ctrl* and *Enter* (Windows & Linux). In a next step (similar to the examples in the section “The paired A–Not A design: Data structure and analysis”), the functions can be used. Entering the given parameters in the brackets and executing the function produces the result in the console (lower part of the window).

The two approaches by Miettinen and Bennet and Underwood reveal similar but not identical results. None of the procedures is more correct than the other since they are based on two different theoretical approaches. In practice, researchers should aim to fulfil the slightly higher values to be on the safe side.

### Practical procedures for the calculations of power and sample size using G*Power

**Fig. 1:**
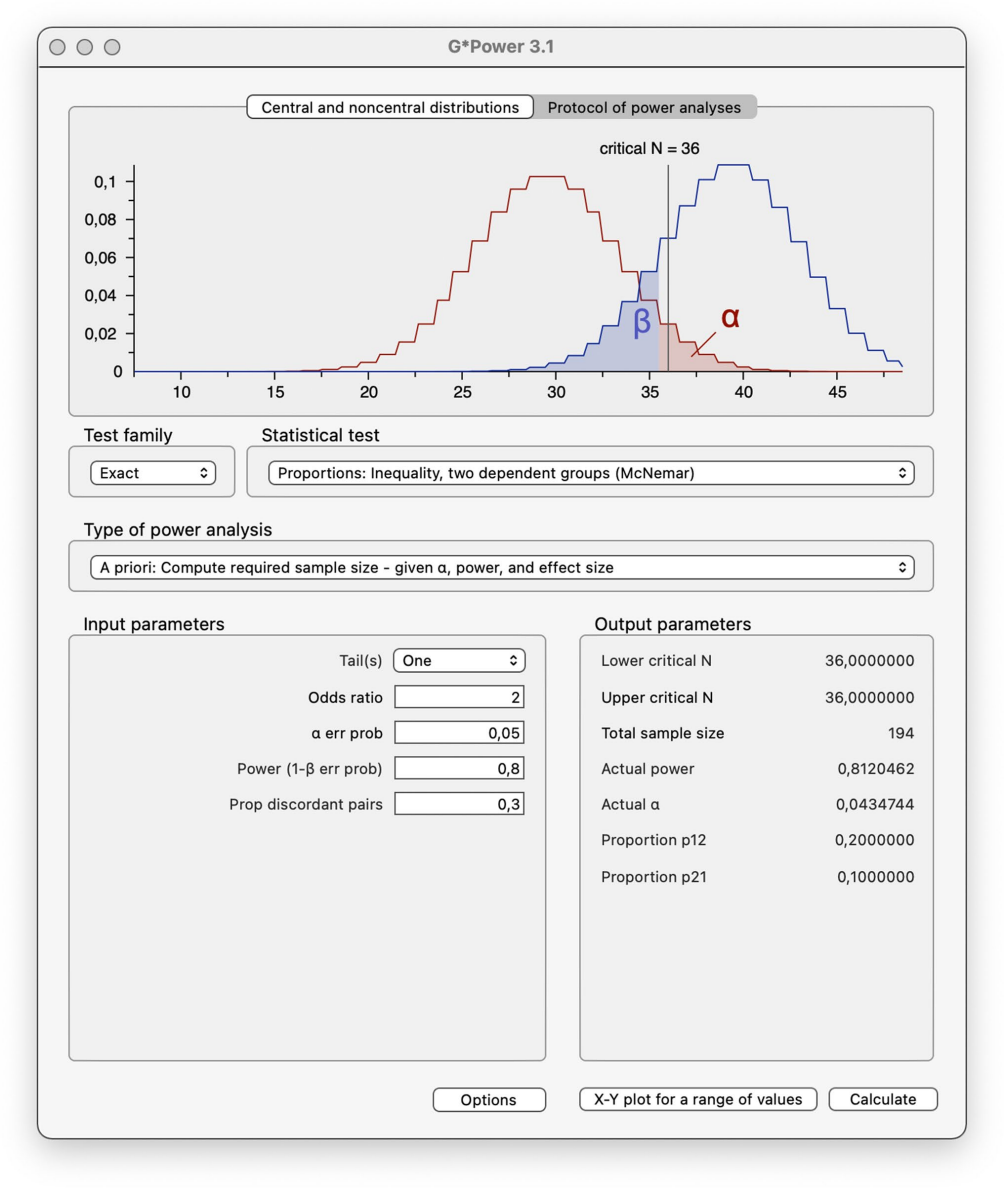
Calculating the required sample size using G*Power

The free software G*Power offers calculations of power and sample size depending on the chosen α-error threshold and the effect size for all common statistical tests. Within the test family Exact, it offers the statistical test Proportions: Inequality, Two Dependent Groups (McNemar) which is the appropriate choice in our case. The function is explained in a paper by Faul et al. (2007, pp. 186–188) and the G*Power manual ("G* Power 3.1 manual", 2017, pp. 14–15). See Fig. 1 for the interface of the software.

The following parameters have to be entered: either a one- or two-tailed test. As the verification of the directed hypothesis is plausible, and the methods described in the sections “Mathematics for the calculation of test power and sample size” and “Practical procedure for the calculation of power and sample size using R” are also one-tailed tests, this option is a reasonable choice. The odds ratio *OR* is calculated according to Eq. 3 in the section “Significance test for the response behaviour”.

Thresholds for the α-error probability and the statistical power (1−β-error probability) must be selected. In this article, the conventional threshold of α-error probability of .05 is used. The threshold for power (1−β-error probability) is set to .8 according to the convention of Cohen (1988, p. 56). The proportion of discordant pairs is also determined by the frequencies *b* and *c* and can be calculated as

9 $${p}_D=\frac{b+c}{N}$$

The button Options reveals possibilities to adjust the alpha balancing in two-tailed tests (described in the "G* Power 3.1 manual", 2017, p. 14) as well as the method of computation. The faster approximation should be used first to get an idea of the magnitude of the required sample size. The exact computation method can be used as a second step but takes considerably more time if the required sample size exceeds approximately *N* = 300. Nevertheless, this exact procedure is of great value for us as it presents an important improvement compared to the approximations from the sections “Mathematics for the calculation of test power and sample size” and “Practical procedure for the calculation of power and sample size using R”. After entering these parameters, a click on Calculate starts the operation, and results are shown in the section “Output parameters”. In our case, the minimum total sample size required is *N* = 194.

### Table of required sample size comparing calculation methods

Table 4 displays required sample sizes for different proportions of *p*_b_ and *p*_c_. The proportions are classified according to their sum, the proportion of discordant pairs *p*_D_ and their odds ratio *OR*. The two approximations from the section “Practical procedure for the calculation of power and sample size using R” (by Bennett and Underwood and by Miettinen) as well as the approximated and the exact method from G*Power (see the section “Practical procedures for the calculations of power and sample size using G*Power”) were used to calculate the required sample sizes for the different *p*_b_ and *p*_c_. All four methods are always listed in one cell of the table and are therefore easily compared. Viewing the sample sizes in the table reveals the basic connections: The smaller the odds ratio *OR* and the smaller the proportion of discordant pairs *p*_D,_ the more participants are required for a study.

## Adjustment from a non-replicated to a replicated paired A–Not A design

### Theoretical considerations and maths

In a non-replicated paired A–Not A design, each participant responds to one (and the same) pair of stimuli in two trials, one per stimulus. In studies in the field of music perception, these stimuli might be two similar musical recordings which participants listen to one at a time and classify in one of the two response categories. The number of response pairs *N* (pairs of data points), therefore, equals the number of participants *n*. If we employ a replicated paired design, each of the *n* participants responds to *k* pairs of stimuli in k*2 trials (*k* = number of replications). The number of response pairs *N,* therefore, results from multiplying the number of stimuli pairs with the number of participants, *N* = *n* ∗ *k*. In practice, the k*2 stimuli are presented in a randomized order to prevent participants from discovering that always to subsequent stimuli are similar and that they likely represent the two stimulus classes. Additionally, we recommend not only relying on randomization but also actively preventing a stimulus from being followed directly by its counterpart. Thereby, one can ensure that participants cannot directly compare the second stimulus to the first from memory. The order of the presentation of the two stimuli of one pair—A first and later Not A or vice versa—makes no difference.

However, this dataset is probably not equivalent regarding statistical power to a setting in which *N* participants evaluated just one pair of stimuli each because the variance between the responses of one participant may be different from the variance in the response patterns between the other participants. If the variances between and within participants differ from each other, the test power is gradually diminished. To obtain the same test power in a replicated design (with sample size *N* = *n* ∗ *k*) as in a non-replicated design (with sample size *N* = number of participants *n*), the number of participants should be corrected upwards in a replicated design. The connection between effective sample size *N*, number of participants *n* and number of replications *k* can be expressed using the adjustment factor *C*:

10 $$N=\frac{n\ast k}{C}$$

*C* can be calculated according to equation 11.2.3 in Bi (2015, p. 305) or with an R script (see the next section, “Practical calculations using R”, in this article). It can show values in a range between 1 and *k* (1 ≤ *C* ≤ *k*). If the calculated value *C* is smaller than 1, *C* = 1 should be assumed (Bi, 2015, p. 316). If *C* = 1 and, therefore, the variance between the answers of participants is the same as within participants, it does not matter whether, for example, 10 participants evaluate 10 pairs of stimuli (resulting in 100 response pairs) or 100 participants evaluate one pair of stimuli each (likewise resulting in 100 response pairs). In this case, *C* can be left out of the equation, and we come back to* N* = *n* ∗ *k*. If *C* > 1, the effective sample size *N* is reduced because of unequal variances. In this case, we would reach less power if we tested 10 participants on 10 stimuli pairs compared to a testing of 100 participants on 1 stimulus pair.

### Practical calculations using R

The adjustment factor *C* as well as the relation between effective sample size *N*, number of participants *n* and number of replications *k* can be calculated using the R script “10 Adjustment from a non-replicated to a replicated paired A–Not A Design.R” from the Supplementary Material. As explained in the previous section, the adjustment factor depends on the variances within and between participants. Therefore, data from a replicated paired A–Not A study should be entered if the researcher wants to calculate the adjustment factor for the specific sample and the given task. The data has to be provided in the structure displayed in Table 3 and saved as a CSV file to be then loaded into the R script. You already calculated the two necessary columns in the Excel file (“4 Calculating the Descriptive Statistics and Effect Sizes.xlsx”) or R script (output is named “7 Calculating the Descriptive Statistics and Effect Sizes_Results from R.csv”) from the section “In practice: Calculating the descriptive statistics and effect sizes”. Now, copy the two columns “Answers ‘A’” and “Answers ‘Not A’” from Excel (second spreadsheet “Data and Calculations”, columns V and W) or columns “Answers_A” and “Answers_Not A” from the CSV-output from R (also columns V and W) into a new table and save as a CSV file. This has already been done for the example data from Düvel et al. (2020) and can be found under the name “9 Sample Data for the Calculation of Adjustment Factor C.csv” in the Supplementary Material. Note that the number of answers “A” is calculated as the sum of Hits and False Alarms and the numbers of answers “Not A” as the sum of Misses and Correct Rejections.

Section 1 in the R script “10 Adjustment from a non-replicated to a replicated paired A–Not A Design.R” defines the function “cbval” which calculates the adjustment factor *C*. First, the code in Section 1 (lines 10 to 48) has to be executed (using *Command* and *Enter* [Mac] or *Ctrl* and *Enter* [Windows & Linux]). Section 2a provides the framework for setting your working directory, uploading your data file (e.g., the sample data “9 Sample Data for the Calculation of Adjustment Factor C.csv”), and calculating *C*. Sections 2b and 2c connect the effective sample size *N* with the number of participants *n* using the calculated adjustment factor *C* and the number of replications *k*. You should always first select the code that defines the function you want to use and execute it. Then, use the function to calculate the parameters for your data.

## Practical implications for the procedure of planning, conducting and evaluating such a study

As presented in the introduction, designs of SDT are frequently employed in studies of psychology, particularly in music psychology. To gain all possible conclusions from the conducted studies and the collected data, you should do a full analysis and not just rely on the report of percentages of correct and wrong answers.

**Fig. 2:**
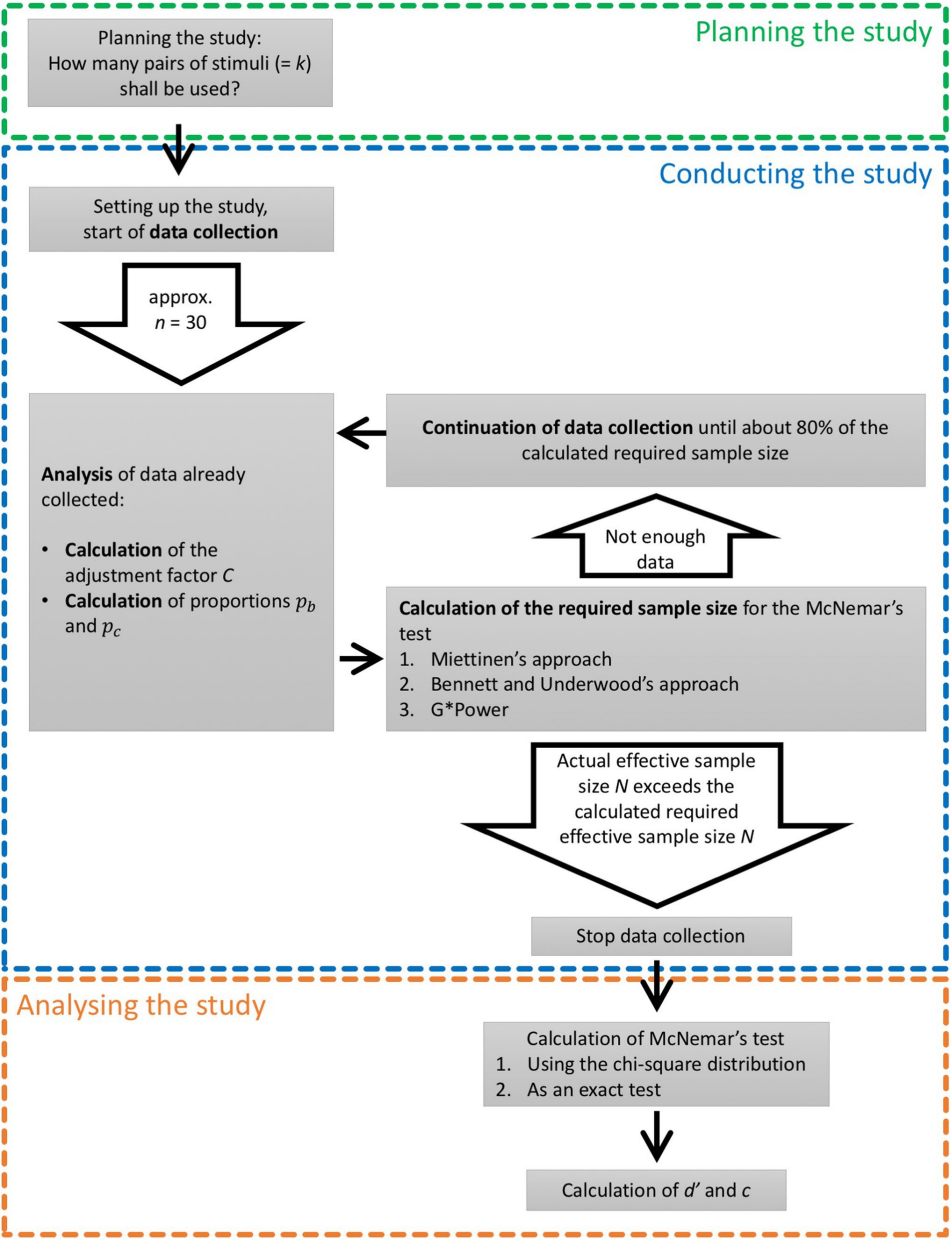
Flowchart for planning, conducting and analysing a study in the replicated paired A–Not A design

This article gives an overview of the different designs within SDT and explains the non-replicated and replicated paired A–Not A designs in detail. The calculation of statistical power is mandatory for a meaningful and conclusive study based on SDT methods, but in contrast to procedures of null hypothesis significance testing (e.g., *t*-test), test power cannot always be conducted before starting the data collection, because information on the proportions *p*_*b*_ and *p*_*c*_ as well as on the adjustment factor *C* is required to conduct a replicated study design. Only if we have a previous study (based on the same methodology and the same topic) that provides us with the required coefficients can we conduct a neat a priori power analysis. In all other cases, the development of a sensible procedure considering the steps of planning a study, collecting data and data analysis is not trivial. Thus, in the final section, we would like to suggest a procedure which has to be reviewed for practicability in future research. Figure 2 sums up all steps of the procedure in a flow chart and will be explained in the following sections.

### Planning the study (before data collection)

When planning a study using the paired A–Not A design, one very important question is: How many pairs of stimuli will be presented to the participants? Several aspects should be considered:

1. The more stimuli you have, the better (see Eq. 10 in the section “Theoretical considerations and maths”). If the adjustment factor *C* of your data is close to 1, it is preferable to have only a few participants who evaluate several pairs of stimuli instead of many participants who are presented with only one or very few pairs of stimuli. However, the validity of this strategy can only be evaluated after calculation of the adjustment factor *C* from the data.
2. How long does it take to evaluate one stimulus? How long is the duration of one stimulus in a design with audio or video examples? In an online study, the total duration of evaluations should not be too long considering that at least some demographic data and/or additional inventories might be of relevance (Reips, 2002). In a lab study, the testing sessions can be longer, but the participants’ concentration should not be overstrained.
3. How many suitable stimuli are available? Depending on the topic and the research question, it might be difficult to organize a large number of adequate stimuli. For example, in the study by Pausch et al. (2021), participants classified musical stimuli based on whether they were performed by a professional musician or by a musical child prodigy. One pair of stimuli consisted of recordings from a professional musician as well as a musical prodigy of the same piece and section of music. One difficulty in designing the study was to find high quality recordings (e.g., studio productions) performed by musical child prodigies, so the possible number of controlled stimuli was very limited. One should always bear in mind that the quality of a study relies on the quality of the stimuli used.

Consideration of these three recommendations will make it more likely to find the appropriate number of stimulus pairs (= *k* = number of replications) for a study. Previous online studies in the domain of music psychology were based on six, 10, and five pairs of carefully selected and constructed stimuli (Düvel et al., 2020; Kopiez et al., 2016; Pausch et al., 2021, respectively).

### Conducting the study (during data collection)

After the start of data collection, data should be analysed, and the preliminary proportions *p*_*b*_ and *p*_*c*_ as well as the adjustment factor *C* should be calculated. This should not result in the practice of collecting data until the results fulfil the hypothesis or the researchers’ wishes (Simmons et al., 2011). Rather, it is advisable to refrain from calculating further statistics as the McNemar’s test and sensitivity *d′* at this point so as not to be (even subconsciously) influenced by the preliminary results. Using *p*_*b*_ and *p*_*c*_, the required effective sample size as outlined in the section “The statistical power of the (non-replicated) paired A–Not A design” can be determined, and after calculating the adjustment factor *C*, the required number of participants *n* can be decided, taking into account the number of replications *k* (which had been decided before starting the data collection). As a rule of thumb, this first analysis could be conducted after collecting data from approximately 30 participants.

Most likely, the calculation of statistical power and required sample size will reveal an insufficient number of data points. Therefore, data collection should be continued until about 80% of the required sample size has been reached. Again, calculations of the proportions *p*_*b*_ and *p*_*c*_ as well as the resulting required effective sample size *N,* the adjustment factor *C*, and the resulting number of participants *n* are repeated. Naturally, the results might have slightly changed because the initial small sample was not representative for the entire sample from the target population.

This circular procedure should be repeated at least one more time to make sure that the collected sample size exceeds the calculated required minimum sample size when all data are considered. After reaching the calculated threshold, data collection can be terminated. The reporting of all descriptive results from calculations related to sample size and statistical power is required.

### Analysing the study (after completing the data collection)

You should calculate McNemar’s test for significant allocation ability (see section “Significance test for the response behaviour”) only after collecting enough data sets. This is followed by the determination of the effect sizes *w*, *d′* and *c* (see section “Effect sizes for the discrimination ability”).

## Summary

The present paper sums up considerations on signal detection theory in general and the non-replicated as well as replicated paired A–Not A design in particular. It not only presents thoughts on the practical application of the design and the subsequent calculation of the sensitivity, for example, but it also addresses the desirable test power and the required minimum sample size. As a suggestion, we describe a step-by-step procedure to guide researchers through the process of planning, conducting, and analysing a study using the paired A–Not A design from the SDT family.

The authors hope that this practically oriented approach might be a significant contribution to the step-by-step development of powerful research designs in future studies and the promotion of methodologically correct and thorough data analysis in empirical research.
